# Mindin deficiency alleviates renal fibrosis through inhibiting NF‐κB and TGF‐β/Smad pathways

**DOI:** 10.1111/jcmm.15236

**Published:** 2020-04-06

**Authors:** Kang Yang, Wei Li, Tao Bai, Yusha Xiao, Weimin Yu, Pengcheng Luo, Fan Cheng

**Affiliations:** ^1^ Department of Urology Renmin Hospital of Wuhan University Wuhan China; ^2^ Department of Anesthesia Renmin Hospital of Wuhan University Wuhan China; ^3^ Department of General Surgery, Research Center of Digestive Diseases Zhongnan Hospital of Wuhan University Wuhan China; ^4^ Wuhan Third Hospital Tongren Hospital of Wuhan University Wuhan China

**Keywords:** inflammatory response, mindin, NF‐κB pathway, renal fibrosis, TGF‐β/Smad

## Abstract

Renal fibrosis acts as a clinical predictor in patients with chronic kidney disease and is characterized by excessive extracellular matrix (ECM) accumulation. Our previous study suggested that mindin can function as a mediator for liver steatosis pathogenesis. However, the role of mindin in renal fibrosis remains obscure. Here, tumour necrosis factor (TGF)‐β‐treated HK‐2 cells and global mindin knockout mouse were induced with renal ischaemia reperfusion injury (IRI) to test the relationship between mindin and renal fibrosis. In vitro, mindin overexpression promoted p65—the hub subunit of the NF‐κB signalling pathway—translocation from the cytoplasm into the nucleus, resulting in NF‐κB pathway activation in TGF‐β‐treated HK‐2 cells. Meanwhile, mindin activated the TGF‐β/Smad pathway, thereby causing fibrotic‐related protein expression in vitro. Mindin^−/−^ mice exhibited less kidney lesions than controls, with small renal tubular expansion, inflammatory cell infiltration, as well as collagen accumulation, following renal IRI. Mechanistically, mindin^−/−^ mice suppressed p65 translocation and deactivated NF‐κB pathway. Simultaneously, mindin disruption inhibited the TGF‐β/Smad pathway, alleviating the expression of ECM‐related proteins. Hence, mindin may be a novel target of renal IRI in the treatment of renal fibrogenesis.

## 1 INTRODUCTION

The incidence and mortality of chronic kidney disease (CKD) continue to increase, severely threatening human health.^1^, ^2^, ^3^ Renal fibrosis, the predominant cause of CKD, is a common pathological outcome in the most advanced patients with CKD.^4^, ^5^ It is characterized by an increased production of extracellular matrix (ECM) proteins including collagen I and fibronectin (Fn).^6^ Excessive deposition of these proteins is considered to contribute to the structural and functional dysfunction of the renal tubule and eventually leads to CKD.^4^, ^7^ Currently, many patients diagnosed with advanced renal fibrosis mainly rely on renal dialysis to maintain their lives because of its irreversibility.^8^ Therefore, antifibrotic treatments that prevent and reverse the progression of renal fibrosis towards CKD are urgently required.

In the progression of renal fibrosis, inflammation provokes a series of detrimental events that leads to renal structure injury and dysfunction.^9^, ^10^ It is well known that nuclear factor (NF)‐κB plays a crucial role in the inflammatory response through up‐regulating the expression of pro‐inflammatory cytokines.^9^ Usually, NF‐κB is latently preserved in the cytoplasm through binding with IκBα inhibitory proteins. During renal fibrosis, IκBα protein is phosphorylated and then releases NF‐κB to enter the nucleus, where it up‐regulates pro‐inflammatory gene expression. It has been proved that the NF‐κB signalling pathway is closely correlated with renal fibrosis.^11^, ^12^ In mice fibrotic kidney, NF‐κB signalling pathway activation up‐regulates tumour necrosis factor (TNF)‐α, interleukin (IL)‐1β and IL‐6, resulting in ECM deposition.^11^ In addition, NF‐κB signalling pathway suppression can attenuate renal fibrosis progress in unilateral ureteral obstruction mice model.

In addition to the NF‐κB pathway, transforming growth factor (TGF)‐β1—the central pathogenic mediator of renal fibrosis—acts via a well‐known canonical pathway that phosphorylates and activates Smad2 and Smad3 by combining with the TGF‐β receptor 1.^13^, ^14^, ^15^ Activated Smad2 and Smad3 then heteroligomerize with conjunct partner Smad4 and this oligomeric complex translocates to the nucleus to regulate targeted gene expressions. Renal tissues from fibrotic patients and mice show higher expression levels of phosphorylated Smad2 (p‐Smad2) and p‐Smad3 compared to those of normal group mice.^16^, ^17^ Smad2 and smad3‐deleted mice had reduced collagen deposition compared to wild‐type mice after unilateral ureteral obstruction, relieving the progression of renal fibrosis.^18^, ^19^ However, activation of the canonical TGF‐β pathway can also promote the expression of inhibitors. Smad7, a negative feedback inhibitor, can block Smad2 and Smad3 access to TGF‐β receptor 1.^13^ Smad7‐deficient mice were more susceptible to renal fibrosis, but Smad7 overexpression could alleviate the fibrotic process in vitro.^20^, ^21^

Mindin (also referred to as spondin 2), a highly conserved ECM protein, is a Spondin2/F‐spondin family member with F‐spondin domains 1 and 2 located at the N‐ and C‐termini as well as thrombospondin type 1 repeats (TSR) at the C‐terminus.^22^ Extensive studies have reported that mindin is an important regulator involved in innate immune response,^23^ liver steatosis and injury,^24^ as well as tumours.^25^ Our previous study also demonstrated that mindin suppresses liver steatosis in mice through interacting with peroxisome proliferator‐activated receptor α (PPARα).^26^ However, how it participates in renal fibrosis, and the relationship between mindin and NF‐κB signalling as well as the TGF‐β1/Smad pathways has not been elucidated.

Based on the aforementioned evidence, we hypothesized that mindin may promote the deleterious progress of kidney fibrosis. Herein, this study proved that in the renal fibrotic mouse model, mindin expression increases in the renal tubular epithelium. Mindin overexpression induces ECM‐related protein expression via activating the NF‐κB and TGF‐β1/Smad pathways, whereas mindin deficiency in mice after renal fibrosis reverses these results. Our study indicates that mindin may serve as a therapeutic target of kidney fibrosis.

## 2 MATERIALS AND METHODS

### 2.1 Mindin knockout mice and genotype

Six‐ to eight‐week‐old breeding pairs of the mindin null C57BL/6J mice (mindin^–/–^) were purchased and maintained in the Center of Experimental Animals of Wuhan University (No. 02103) under specific pathogen‐free conditions. Age‐ and sex‐matched mindin^–/–^ C57BL/6 male mice were subjected to renal fibrosis models. Mindin deficiency was ascertained by real‐time polymerase chain reaction (RT‐PCR).

### 2.2 Animal models

Mouse models of renal fibrosis were constructed by renal ischaemia reperfusion injury (IR) as described previously.^27^, ^28^ Briefly, male C57BL/6 mice (20‐25 g, 8‐10 weeks) were obtained from the Center of Experimental Animals, Wuhan University (Hubei, China). The mice were randomly divided into four groups (n = 5 per group): (a) sham operated, (b) wild‐type renal IRI, (c) mindin^–/–^ sham operated and (4) mindin^–/–^ renal IRI. After general anaesthesia with 50 mg/kg pentobarbital, bilateral kidney pedicels were exposed and clamped by Atraumatic Schwartz microvessel clamp for 45 minutes. Then, the clamps were removed for reperfusion until normal colour was restored. The sham group mice were subjected to the same operation without pedicel clamping. All mice were sacrificed after eight weeks and kidneys were collected for further investigation. Serum was harvested from mouse eyeball blood. Mindin was tested by enzyme linked immunosorbent assay (ELISA) kit (LifeSpan BioSciences (LS‐F14425) according to the manufacturer's protocol. All animal experiments were performed according to the guidelines of the Institutional Animal Care and Use Committee of Renmin Hospital of Wuhan University and the Animal Ethics Committee at the Renmin Hospital of Wuhan University.

### 2.3 Cell culture and treatment

Normal human kidney proximal tubule epithelial cell line (HK‐2) was purchased from China Center for Type Culture Collection (Wuhan, China) and cultured according to the instructions. HK‐2 cells were seeded into six‐well plates and maintained in complete medium overnight. Then, the cells were incubated in the complete medium supplemented with polybrene (Sigma, USA) and transfected with lentivirus (GeneChem, China), pLVX‐HK‐2‐Mindin‐ZsGreen‐Puro (pLV‐Mindin) and pLVX‐HK‐2‐ZsGreen‐Puro (pLV‐Vector) according to the manufacturer's protocol. After 24 hours, the cells were cultured in complete medium supplemented with 10% foetal bovine serum (FBS, ScienCell, USA). Finally, the cells were photographed under a microscope and the transfection was verified by RT‐PCR and Western blot.

For renal fibrosis in vitro, 1.5 × 10^6^ cells were seeded into six‐well plates and maintained in complete medium overnight. After starvation for 12 hours without 10% foetal bovine serum, HK‐2 cells at 60%‐70% concentration were treated with human recombinant TGF‐β1 (R&D, USA) at a final concentration of 20 ng/mL. Then, the cells were harvested for further analysis after 24 hours.

### 2.4 Histology and immunohistochemistry assay

Four‐μm thick sections from paraffin‐embedded mouse kidney were stained with haematoxylin and eosin (HE), Sirius Red Stain and Masson's trichrome Staining (MTS) to evaluate kidney structure and fibrosis injuries based on the manufacturers’ instructions. Tubular injury score was graded from 0 to 4 based on the cortico‐medullary region percentage as previously described^29^: 0, no damage; 1, <25%; 2, 25%‐50%; 3, 50%‐75%; and 4, >75%. The fibrotic positive area of the murine kidney was determined by calculating the percentage of colour‐pixel count in the entire field containing the cortico‐medullary region. The renal injury and fibrotic area were evaluated by two pathologists and five randomly selected fields of each slide were quantified at 200× magnification. Histology and immunohistochemistry (IHC) staining was performed according to the manufacture's protocols as previously described. Briefly, each slide from the sample was deparaffinized and the antigen was retrieved. After incubating with antibodies (Table S1) overnight at 4°C, each tissue was incubated with horseradish peroxidase‐conjugated secondary antibody and stained with 3,3‐diaminobenzidine tetrahydrochloride (DAB, Maixin, China). The positive area of each slide was photographed under a microscope (Olympus, Japan) at 200× magnification in five random fields, and the results were analysed by Image Pro Plus 6.0.

### 2.5 Immunofluorescence staining

Paraffin‐embedded murine kidney sections and cells cultured on coverslips were prepared by routine protocols and then incubated with primary antibodies (Table S1) at 4°C for 12 hours. Subsequently, the slides were immunostained with Alexa Fluor 488‐ and 555‐conjugated secondary antibodies (1:200; Cell Signaling Technology, USA), and the nuclei were stained with 4′,6‐diamidino‐2‐phenylindole. Images of each slide were viewed by fluorescence microscope (Olympus, Japan).

### 2.6 RNA isolation and real‐time quantitative polymerase chain reaction (RT‐qPCR) Assays

Total RNA was extracted from murine kidneys with Trizol reagent (Thermo Fisher Scientific) and cDNA was synthesized using with a SuperScript cDNA Synthesis Kit (Thermo Fisher Scientific) according to the protocols given by the manufacturer. RT‐qPCR was performed with SYBR Green‐based reagent (Qiagen, USA) on StepOnePlus™ Real‐Time PCR System (Applied Biosystems). The primer sequences used in this study are as follows:

### 2.7 Western blot

Cells and kidneys of the mouse were lysed with Lysis Buffer (Servicebio, China) containing phosphatase inhibitors and protease inhibitor cocktail (Thermo Fisher Scientific) and the protein concentration was measured using BCA Protein Assay Kit (Thermo Fisher Scientific). A total of 20 to 30 μg of each protein were separated using 10% sodium dodecyl sulphate‐polyacrylamide gel and then transferred to a polyvinylidene difluoride membrane. The membranes were blocked with 5% milk for 1 hour and incubated with primary antibodies (Table S1). The blots were scanned with two‐colour infrared imaging system (Odyssey, LI‐COR, USA) and analysed by ImageJ software. Nuclear and cytoplasmic extraction reagent (Thermo Fisher Scientific) was utilized to dissociate the nuclear and cytoplasmic proteins according to manufacturer's instructions.

### 2.8 Statistical analysis

All results are presented as mean ± SEM. All experiments in our study were plicated three times. The results of two groups were compared using Student's *t* test and multiple groups were compared using one‐way ANOVA with Bonferroni post hoc test conducted by SPSS 16.0 software (Chicago, USA). *P* < .05 was considered statistically significant.

## 3 RESULTS

### 3.1 Mindin levels are increased in murine fibrotic kidneys

**FIGURE 1:**
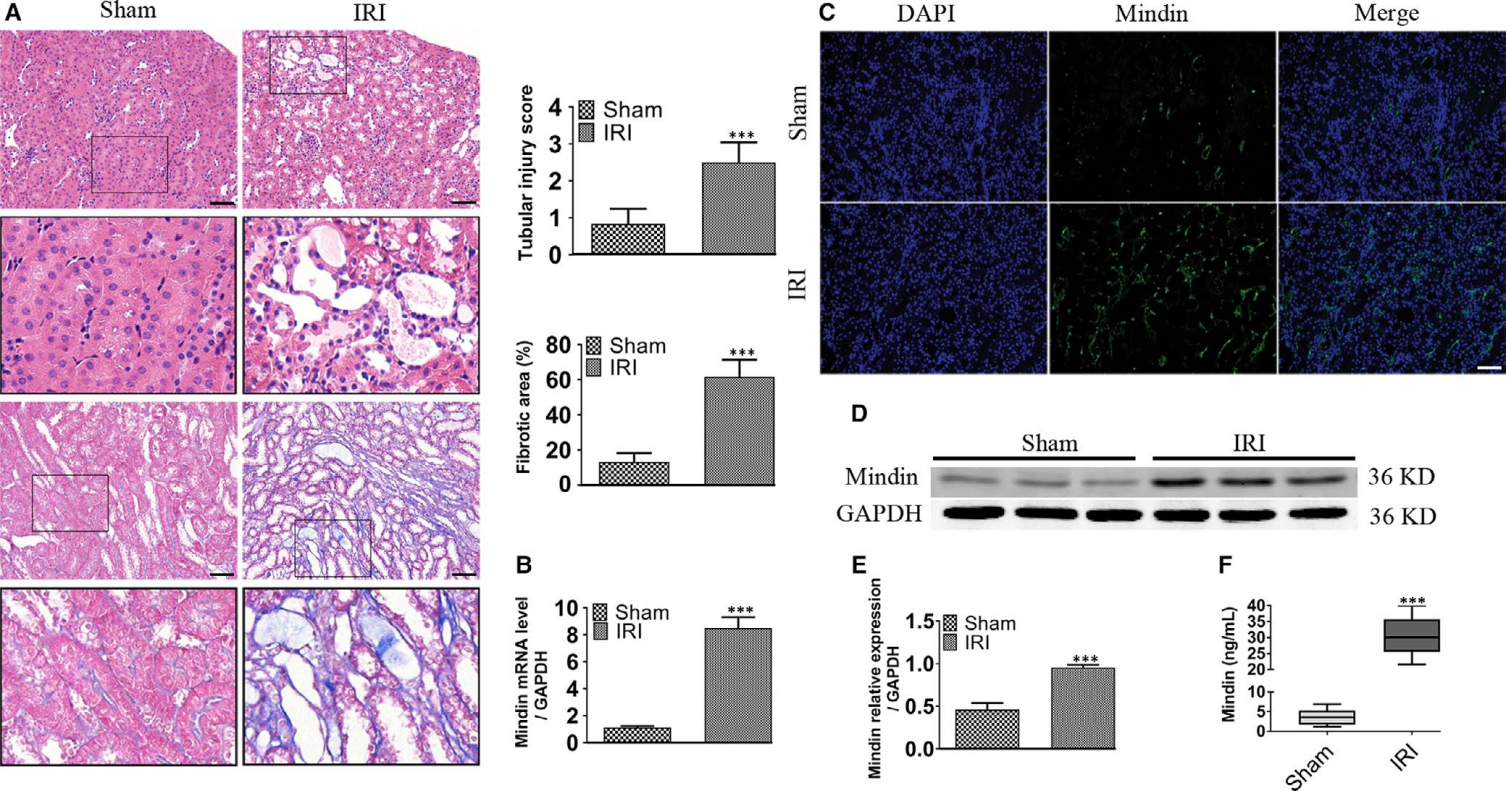
Mindin is induced in murine fibrotic kidney. (A) Representative micrographs and quantification analysis of haematoxylin and eosin and Masson's trichrome staining of sham group and eight‐week renal ischaemia reperfusion injury (IRI) mice. Magnification, 200×. Scale bar, 50 µm. Quantification analysis of the tubular injury and fibrotic score in each group as described above. (B) Real‐time qPCR analysis of mindin following IRI. (C) Representative immunofluorescence staining of Mindin in sham group and IRI group. Green fluorescence intensity indicates mindin‐positive renal tubules. Magnification, 200×. Scale bar, 50 µm. (D) Western blot and (E) quantitative analysis of mindin protein in sham and IRI kidneys. (F) Serum level of mindin from mice was detected using an ELISA. Wilcoxon two‐sample test was used to evaluate the differences between the sham and renal IRI groups. Data are presented as the mean ± SE (n = 5 mice per group). ***P < .001 versus sham group

To explore mindin expression in regulating fibrosis, we first studied the fibrotic models induced by renal IRI. As is shown in Figure 1A, the IRI group mice had severe tubular damage including renal tubular expansion, intertubular haemorrhaging and massive deposition of collagens, suggesting that the model of IRI was well established. The result of RT‐qPCR analysis revealed that mindin mRNA levels in IRI group mice were markedly increased than those in sham group mice (Figure 1B). Consistently, immunofluorescence staining showed that mindin expression was significantly elevated than that in sham controls and was mainly located in the expanded renal tubule of fibrotic kidneys (Figure 1C). Similarly, mindin levels were also increased in murine fibrotic kidneys, as proved by Western blot (Figure 1D and E). Additionally, the results of ELISA showed that the levels of mindin were remarkably increased in the serum of mice after renal fibrosis (Figure 1F). These results suggest that mindin is an inducible renal tubule‐derived protein that may play an important role in murine renal fibrosis pathogenesis.

### 3.2 Mindin aggravates inflammation through NF‐κB signalling pathway in vitro

**FIGURE 2:**
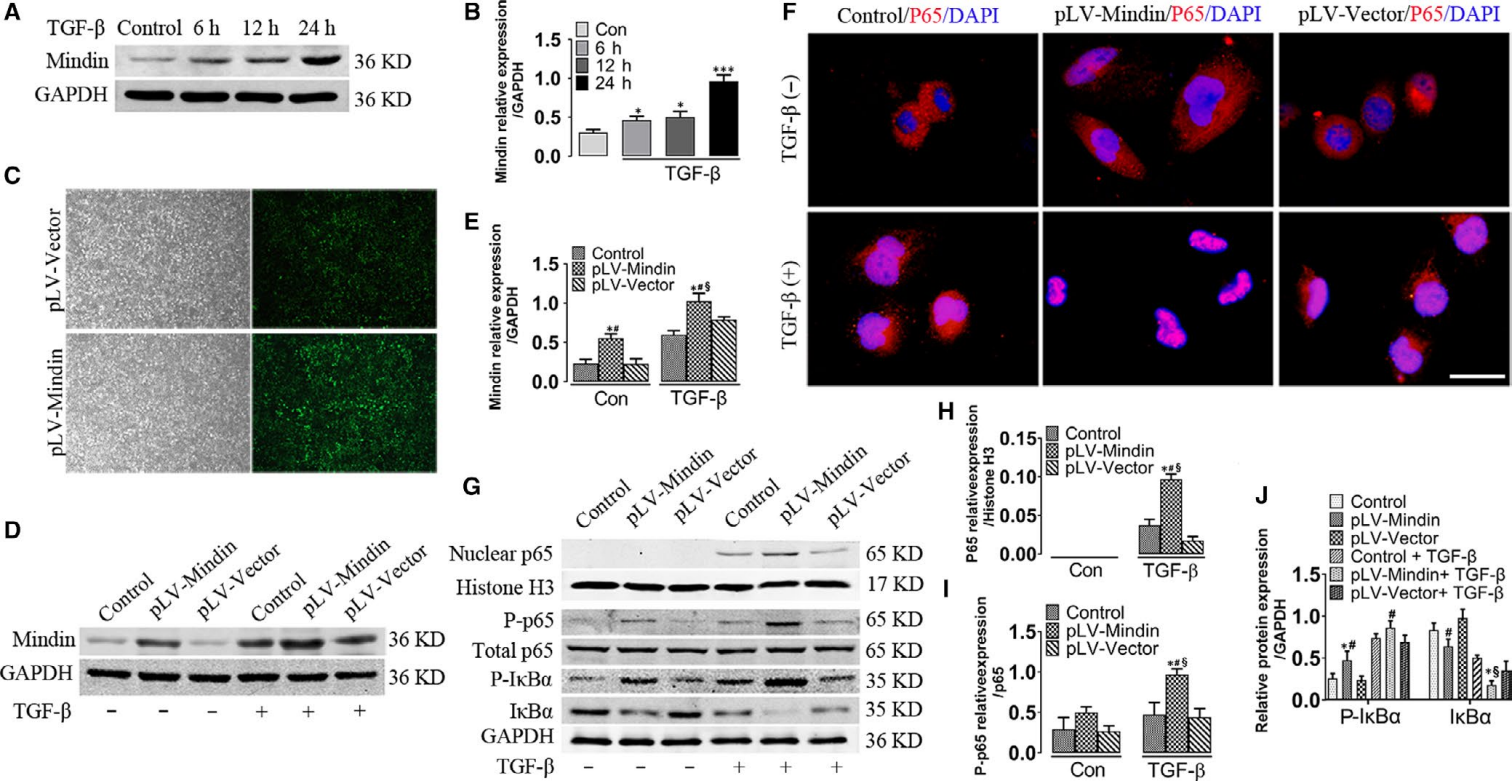
Mindin promotes inflammation via regulating the NF‐κB signalling pathway. (A) Western blot and (B) quantitative analysis of mindin expression in HK‐2 cells after treating with TGF‐β for 6 h, 12 h, 24 h and control group. (C) Representative micrographs showing the immunofluorescence of green fluorescent protein in pLV‐Vector and pLV‐mindin groups. Magnification, 400×. Scale bar, 10 µm. (D) Western blot and (E) quantitative analysis of mindin expression in control, pLV‐Vector and pLV‐mindin groups. (F) Immunostaining for p65 was performed with HK‐2 cells of all groups. Magnification, 1000×. Scale bar, 20 µm. (G) Western blotting analysed protein expressions of p65 nuclear fraction and p65 total lysate, phosphorylated‐p65 (p‐p65), IkBα and phosphorylated‐IkBα (p‐IkBα) of HK2 cells lysates. Histone H3 and GAPDH were respectively used as loading controls of nuclear and total proteins. (H) Densitometric analysis for the expression of p65 nuclear proteins levels in HK2 cells. (I–J) Quantification of NF‐κB signalling pathway‐related proteins levels in total HK2 lysates. Data are presented as the mean ± SE. *P < .05 versus control, # *P* < .05 versus pLV‐Vector group, § *P* < .05 versus pLV‐Mindin group

Inflammatory response has been reported to correlate with renal fibrosis in CKD pathogenesis.^30^ Therefore, immunofluorescence and Western blot analysis were performed to investigate whether mindin could activate NF‐κB signalling pathway, the most important inflammation regulator, in vitro. First, Western blot was utilized to verify the level of mindin in HK‐2 cells treated with different concentrations of TGF‐β (Figure 2A). The results indicated that mindin expression gradually increased and was markedly elevated at 24 hours (Figure 2B). Meanwhile, mindin was stably overexpressed in HK‐2 cells (Figure 2C) and Western blot analysis detected transfection efficiency (Figure 2D and E). Immunofluorescence staining of p65 displayed that p65 was translocated from the cytoplasm of HK‐2 cells into the nucleus in response to TGF‐β stimulation, and the p65 expression in the pLV‐Mindin group cells was increased compared to that in pLV‐Vector and control group cells after treatment (Figure 2F, red colour). Similarly, Western blots of nuclear proteins in HK‐2 cells analysis indicated that mindin overexpression significantly enhanced p65 expression in the pLV‐Mindin group cells with stimulus than that in control and pLV‐Vector group cells (Figure 2G and H). These results suggest that the NF‐κB signalling pathway was activated and may be linked with mindin in renal fibrosis in vitro. To elucidate the underlying effect of the NF‐κB pathway, the crucial proteins involved were detected by Western blot. As shown in Figure 2G, I and J, p65, phosphorylated‐p65 (p‐p65), and phosphorylated‐IκBα (p‐IκBα) protein levels were increased and IκBα expression was decreased in TGF‐β‐treated pLV‐Mindin group cells compared to that in TGF‐β‐untreated pLV‐Mindin group cells. Moreover, compared with those in the TGF‐β‐treated pLV‐Vector group cells, mindin overexpression significantly increased the p‐p65 and p‐IκBα protein levels and attenuated IκBα levels in TGF‐β‐treated pLV‐Mindin cells. Our data suggest that mindin might be capable of exacerbating inflammation in part through activating the NF‐kB signalling pathway.

### 3.3 Mindin promotes matrix production via activating TGF‐β/Smad pathways

**FIGURE 3:**
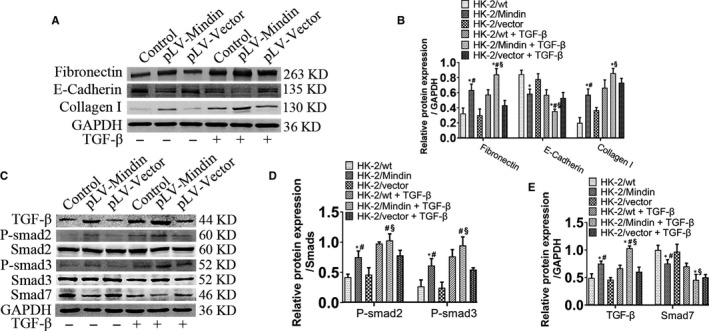
Mindin overexpression facilitates renal fibrosis by affecting the TGF‐β/Smad signalling pathway in vitro. (A) Western blotting analysed protein expressions of fibronectin, E‐Cadherin and collagen I. GAPDH was used as a loading control. (B) Quantification analysis of these protein levels. (C) Western blotting analysis of the TGF‐β/Smad signalling pathway‐related proteins, and quantification of these proteins’ levels. (D and E) Data are presented as the mean ± SE. *P < .05 versus control, # *P* < .05 versus pLV‐Vector group, § *P* < .05 versus pLV‐Mindin group

To define the underlying effect of mindin on renal fibrosis using an in vitro model, Western blot was performed to test the expression levels of ECM‐related proteins like Fn, E‐Cadherin and collagen I (Figure 3A). TGF‐β significantly increased Fn and collagen I protein levels whereas decreased E‐Cadherin expression compared to that in the untreated groups. Fn and collagen I expressions increased while E‐Cadherin levels decreased in the pLV‐Mindin group cells compared to that in the control group cells (Figure 3B). To further determine potential action mechanisms, TGF‐β/Smad pathway‐related proteins were examined by Western blotting (Figure 3C). As expected, compared with those in pLV‐Mindin group cells, TGF‐β, p‐Smad2 and p‐Smad3 expressions were drastically increased; in contrast, Smad7 levels were decreased post‐stimulation. Furthermore, these regulating trends were seen between the pLV‐Mindin and control group cells (Figure 3D and E), suggesting that mindin may be capable of inducing pro‐fibrotic matrix deposition through up‐regulating TGF‐β/Smad pathway‐related proteins in HK‐2 cells incubated with TGF‐β recombinants.

### 3.4 Genetic ablation of mindin attenuates renal fibrosis after renal IRI

**FIGURE 4:**
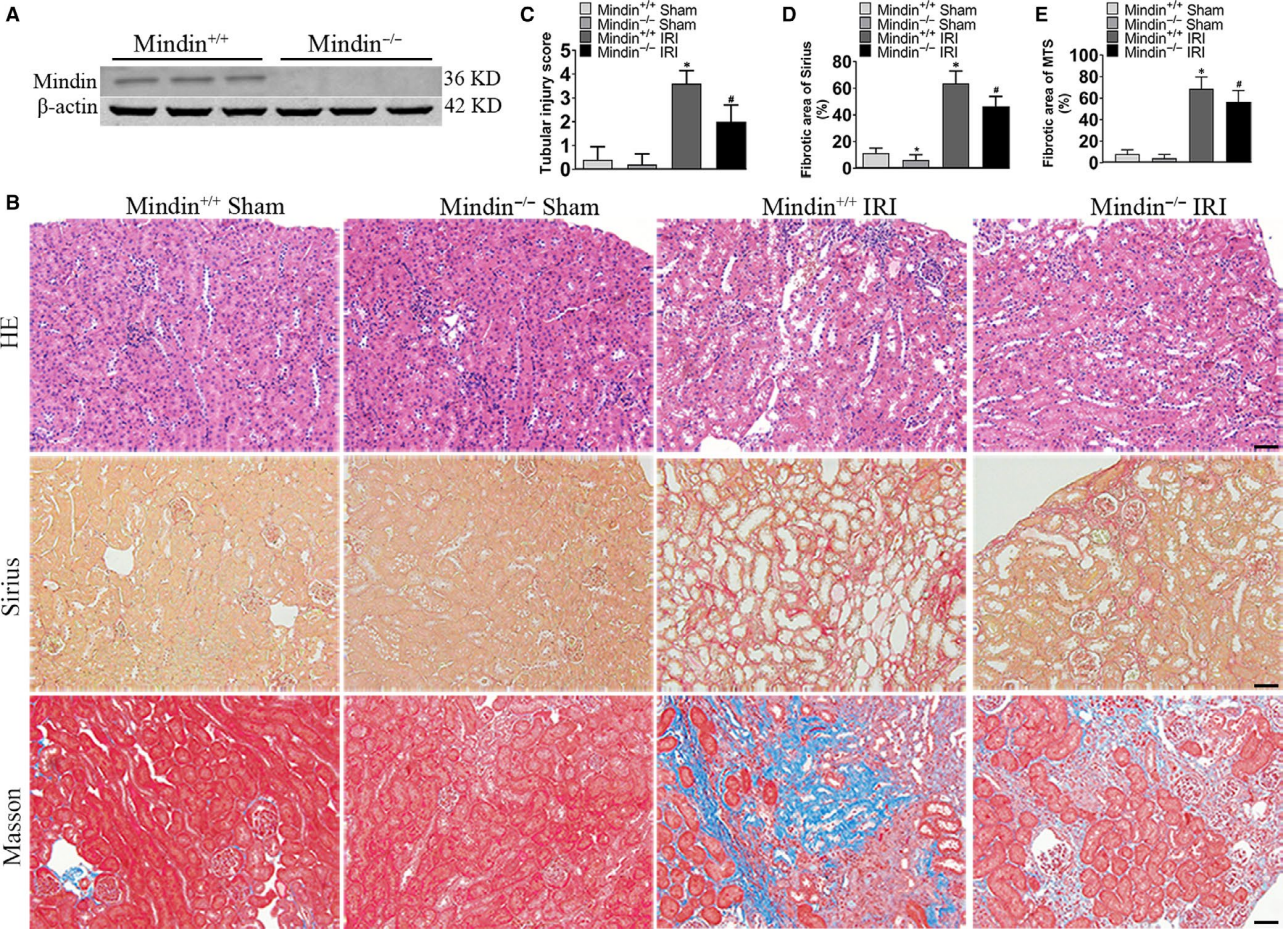
Mindin deficiency reduces renal fibrosis after renal ischaemia reperfusion injury in vivo. (A) Western blot analysis of mindin expression in wild‐type (WT) and mindin knockout (KO) mice. (B) Representative micrographs of HE, Sirius red and Masson's staining (MS) of mindin^+/+^ and mindin^−/−^ kidneys with or without renal ischaemia reperfusion injury. Magnification, 200×. Scale bar, 50 µm. (C–E) Quantitative determination of renal injury score and pro‐fibrotic area in KO and WT mice. Data are presented as the mean ± SE (n = 5 mice per group). *P < .05 versus sham WT mice, # *P* < .05 versus WT mice after renal IRI

To verify the in vitro findings, mindin null mice (mindin^–/–^) were utilized to investigate whether mindin loss protected kidneys against renal fibrosis after IRI (Figure 4A). HE, Sirius red staining, and MTS were performed to reveal that compared with mindin^+/+^ mice, mindin^–/–^ mice exhibited no morphological abnormality and fibrotic area alteration (Figure 4B). Disruption of mindin in renal fibrosis group mice exhibited less renal tubular expansion, inflammatory cell infiltration and collagen accumulation compared with those in the renal IRI‐induced obstructed kidneys in wild‐type mice (Figure 4B). In addition, compared with that in the wild‐type group mice, the renal injury score was significantly reduced in mindin deficiency group mice after renal IRI (Figure 4C). Similarly, mindin ablation remarkably reduced the renal interstitial fibrosis area compared with that in the wild‐type controls (Figure 4D and E).

### 3.5 Mindin deficiency alleviates inflammation through inhibiting NF‐κB signalling pathway in vivo

**FIGURE 5:**
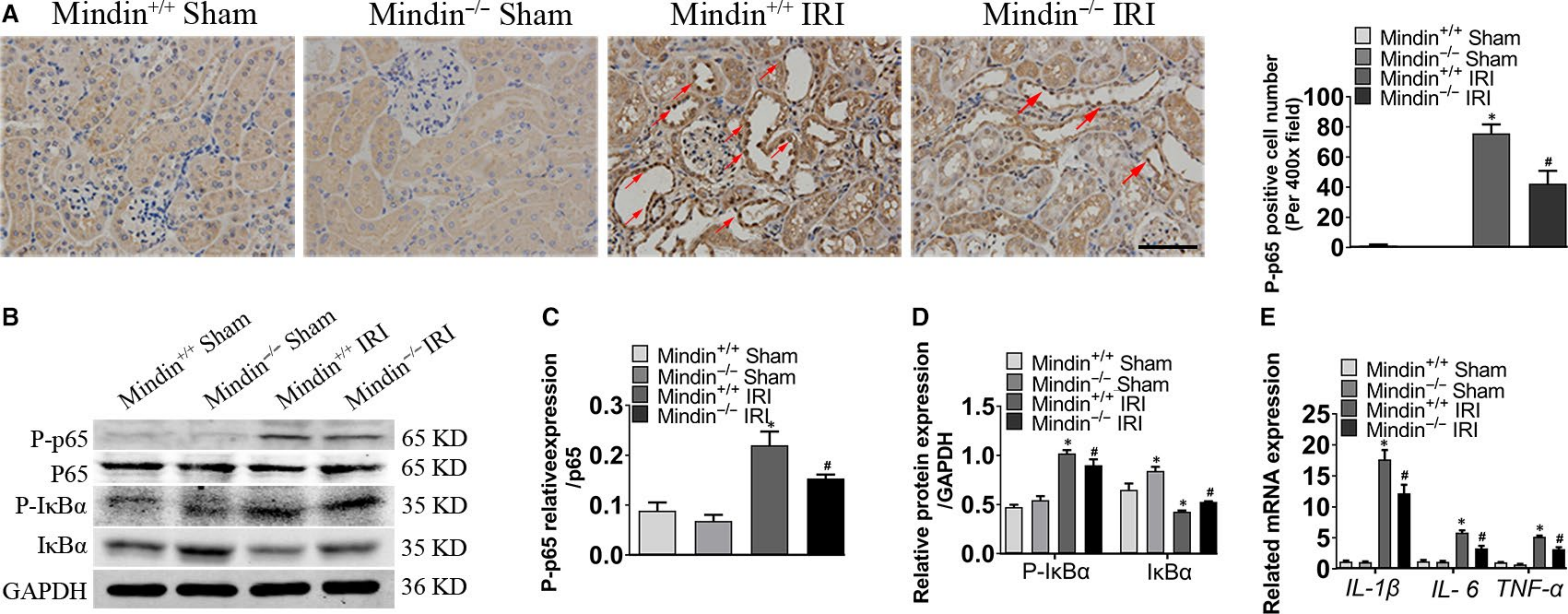
Disruption of mindin attenuates inflammation in obstructive kidneys. (A) Representative micrographs and quantitative analysis of p‐p65 immunostaining in wild‐type and mindin^−/−^ mice with or without renal IRI. Red arrows denote positive staining of p65. Magnification, 400×. Scale bar, 50 µm. (B) Western blotting analysis of protein expressions of p65, p‐p65, IkBα and p‐IkBα in mindin^+/+^ and mindin^−/−^ kidneys with or without renal IRI. GAPDH was used as a loading control. (C and D) Quantification of NF‐κB signalling pathway‐related protein levels. (D) RT‐qPCR analysis of inflammatory cytokines *IL‐1β, IL‐6* and *TNF‐α* in the indicated groups. Data are presented as the mean ± SE, n = 5 mice per group. *P < .05 versus sham WT mice, # *P* < .05 versus WT mice after renal IRI

Inflammation is an important feature of kidney lesions in renal fibrosis after renal IRI. Therefore, to explore the potential effect of mindin on inflammation in renal fibrosis in vivo, mindin^–/–^ mice were utilized to determine whether mindin suppressed the crucial NF‐κB signalling pathway of inflammation. As shown in Figure 5A, compared with that in the wild‐type controls, p‐p65 expression was dramatically increased in the obstructed kidneys after renal IRI and the expanded renal epithelial cell nuclei were stained with p65 (Figure 5A, red arrows), suggesting that the NF‐κB pathway was activated, while mindin loss alleviated p65 induction. To investigate the underlying mechanism, Western blot analysis of whole kidney lysates was performed to further examine NF‐κB pathway‐related proteins (Figure 5B). As indicated in Figure 5C and D, renal IRI resulted in significant increase of p‐p65 and p‐IκBα expressions, while IκBα expression was decreased compared with that in the sham group mice. However, mindin ablation reduced this regulating effect. Interestingly, the expressions of pro‐inflammatory genes *IL‐1β*, *IL‐6* and *TNF‐α* as detected by RT‐qPCR were in line with the p65 immunostaining results. These data suggest that mindin plays an important role in NF‐κB signalling pathway‐induced inflammation after renal IRI.

### 3.6 Loss of mindin reduces the levels of ECM via suppressing TGF‐β/Smad pathway after renal IRI

**FIGURE 6:**
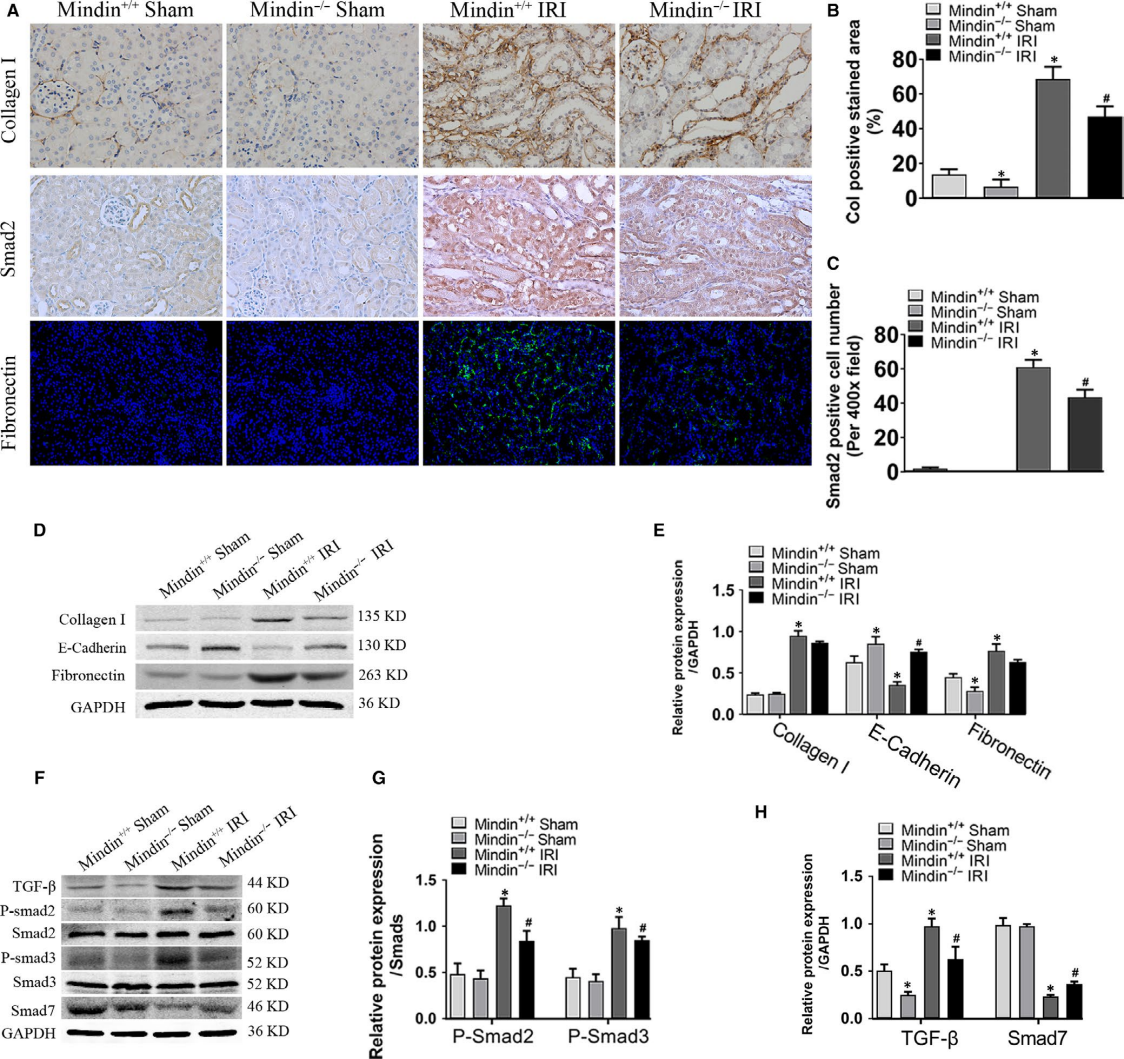
Mindin knockout reduced pro‐fibrotic protein expression though inhibiting TGF‐β/Smad signal pathway activation in mice after renal ischaemia reperfusion injury. (A) Representative micrographs of collagen I (Col), Smad2 and fibronectin (Fn) immunohistostaining (IHC) and Fn immunofluorescence staining in wild‐type and mindin^−/−^ mice with or without renal IRI. Magnification, 400×. Scale bar, 50 µm. (B and C) Quantitative analysis of IHC with Col and Smad2 in mindin ^+/+^ and mindin^−/−^ mice with or without renal IRI. (D) Western blotting analysis of extracellular matrix‐related proteins fibronectin, E‐Cadherin and Col. GAPDH was used as a loading control. (E) Quantification analysis of these proteins’ levels. (F) Western blotting analysis of TGF‐β, p‐Smad2 and p‐Smad3 and Smad7 proteins. GAPDH was used as a loading control. (G and H) Quantification analysis of the proteins’ expressions. Data are presented as the mean ± SE, n = 5 mice per group. *P < .05 versus sham WT mice, # *P* < .05 versus WT mice after renal IRI

To understand whether mindin could alleviate the kidney lesions in the progress of renal fibrosis, we then examined the ECM‐related proteins in vivo. The results of IHC staining showed that renal IRI significantly induced type 1 collagen expression in wild‐type mice and mindin knockout relieved these inductions (Figure 6A and B). Interesting, similar results were obtained by immunofluorescence analysis with fibronectin (Figure 6A). To further prove these findings, Western blot analysis with ECM‐related proteins like fibronectin, collagen I and E‐Cadherin was performed (Figure 6D). As indicated in Figure 6E, compared with those in the sham group mice, fibronectin and collagen I expressions were dramatically increased in wild‐type mice and the level of E‐Cadherin was remarkably reduced after renal IRI, while ablation of mindin attenuated these alterations induced by renal IRI.

Many studies have suggested that TGF‐β/Smad pathway activation plays a crucial role in the progress of renal fibrosis.^13^, ^14^, ^31^ To test this possibility and further confirm the in vitro findings above, we then examined TGF‐β/Smad pathway‐related proteins in vivo (Figure 6F). IHC analysis showed that renal IRI promoted Smad2 translocation from the cytoplasm to the nucleus in the mindin^+/+^ group mice compared with that in sham wild‐type mice, and mindin knockout decreased Smad2 expression compared with that in control group mice after renal IRI (Figure 6A). A significant increase in TGF‐β, p‐Smad2, as well as p‐Smad3 expressions and a remarkable decrease in Smad7 expression were discovered in the obstructed kidney of wild‐type mice after renal IRI. Moreover, mindin loss attenuated TGF‐β, p‐Smad2, as well as p‐Smad3 expressions and promoted Smad7 protein level compared with that in sham group mice after renal IRI. These results suggest mindin can reduce ECM‐related proteins production through suppressing TGF‐β/Smad pathway in the murine kidney after renal IRI.

## 4 DISCUSSION

Renal tubulointerstitial fibrosis is considered as a poor prognostic indicator for progression of CKD and represents pathological features that result in the derangement of renal structure and irreversible functional loss of kidney.^32^, ^33^ Nowadays, patients with CKD are usually diagnosed by kidney biopsy, which is an invasive procedure and is more inconvenient and even painful for patients.^34^, ^35^ Therefore, it is imperative to elucidate the pathogenesis of kidney fibrosis and develop a biomarker or therapeutic target to prevent patients from contracting CKD.

Previous research has investigated the function of mindin in a series of diseases such as cardiac hypertrophy,^36^ liver injury,^24^ as well as diabetic nephropathy.^37^ Our previous study proved that mindin correlates with hepatic lipid metabolism via mediating the PPARα signalling pathway. Consistent with the results of previous studies, we revealed that the levels of mindin were increased in renal fibrotic kidney and serum. Mindin is a kinds of extracellular matrix proteins. During the occurrence of renal fibrosis, cells secrete extracellular matrix proteins after stimulation. These proteins are deposited in extracellular area and the large amount of matrix produced increases the high osmotic pressure between cells, thereby absorbing fluid of cells. Interstitial fluid, which eventually flows into the blood, causing an increase in extracellular matrix protein levels in the blood.^38^, ^39^ The potential mechanisms of mindin in renal fibrosis were further investigated in vitro and in vivo.

Injured renal tubular epithelial cells induce an inflammatory response through releasing a series of pro‐inflammatory cytokines such as interleukin and tumour necrosis factor, which is an important pathological feature in the progress of renal fibrosis after renal IRI.^40^ It is well established that the NF‐κB pathway plays a crucial role in regulating inflammation. The hub subunit p65 is normally deactivated by interacting with IκBα in the cytoplasm. After being stimulated, IκBα is phosphorylated and degraded by the ubiquitin enzyme, leading to p65 translocation to the nucleus to regulate pro‐inflammation.^9^ It has been reported that mindin correlates with this inflammatory response through recruiting inflammatory cells.^41^ High levels of mindin interact with receptors in neutrophils and macrophages, thereby providing adhesion sites for their migration and consequent inflammatory action.^41^ As illustrated by our results, inflammatory cytokines IL‐1β, IL‐6 and TNF‐α were up‐regulated in fibrotic kidneys, and mindin ablation down‐regulated these expressions. These suggest that mindin may promote inflammatory cytokine release from inflammatory cells, leading to kidney tissue injury. In the murine stroke models, mindin ablation could suppress cerebral ischaemia reperfusion‐induced inflammation through inhibiting NF‐κB pathway activation.^42^ Consistent with these findings, present research indicates that mindin overexpression promotes p65 translocation from the cytoplasm into the nucleus, leading to NF‐κB pathway activation in vitro, while mindin disruption deactivates the NF‐κB pathway in mice after renal IRI; this suggests that mindin might exacerbate renal damage, at least partially, through regulating the NF‐κB signalling pathway in the progress of renal fibrosis.

Apart from inflammation, ECM production and deposition have also been considered as important pathological events during renal fibrosis.^43^, ^44^ Excessive ECM‐related proteins such as Fn, E‐Cadherin and collagen I result in fibrotic scar formation as well as renal parenchymal lesion, and finally renal tissue remodelling. Increased Fn and collagen I levels, expressed mainly by myofibroblasts, initiate renal parenchyma destruction; collagen I overexpression induces E‐Cadherin down‐regulation, leading to renal tubular epithelial cells losing their adhesive capacity and acquiring mesenchymal features.^45^ In the present study, the expressions of fibrotic‐related proteins collagen I and Fn were increased in HK‐2 cells and murine kidneys induced by fibrosis. Interestingly, overexpression of mindin in cells further accelerated these changes but mindin deficiency attenuated the alterations. Meanwhile, E‐Cadherin expression was decreased in TGF‐β‐administered pLV‐Mindin group cells compared with that in the control group cells. However, mindin‐deficient mice displayed elevated E‐Cadherin expression compared with the wild‐types. These data prove that mindin exhibits an important effect in regulating fibrotic proteins.

The TGF‐β/Smad pathway has been demonstrated to act as a central renal fibrosis mediator in numerous animal and human studies.^2^, ^13^, ^14^, ^40^ Activated TGF‐β conveys intracellular signals by phosphorylating Smad2 and Smad3, rendering downstream fibrotic protein expression, and inhibiting the degradation of these proteins.^16^ Present results, therefore, support the notion that TGF‐β/Smad‐related proteins p‐Smad2 as well as p‐Smad3 are up‐regulated, whereas Smad7 expression decreased in renal fibrosis models compared with that in the controls. The expressions of fibrotic proteins were in correlation with the trends of the TGF‐β/Smad pathway. Interestingly, both genetic knockout mindin mice and HK‐2 cells with stably overexpressed mindin suppressed and promoted the levels of TGF‐β/Smad signalling pathway‐ and targeted‐related proteins, respectively, suggesting that mindin might exacerbate renal damage through activating the TGF‐β/Smad signalling pathway and the downstream targeted pro‐fibrotic proteins during renal fibrosis.

However, there are some limitations to our study. During the early stages of renal fibrosis, continuous inflammatory response of renal tubular cells and immune cells promotes TGF‐β/Smad pathway activation and overexpressed Smad2 and Smad3 further activate the NF‐kB pathway in turn.^11^, ^46^ Nevertheless, at the middle and late stages, Smad7, the inhibitory Smad in TGF‐β/Smad pathway, is capable of promoting IkBα expression, resulting in NF‐kB pathway deactivation.^47^ Our results illustrated that both the NF‐kB and TGF‐β/Smad pathways were activated by mindin during renal fibrosis, which was consistent with previously reported results.^11^, ^21^ However, the relationship between mindin and the two pathways, and how mindin regulates the pathways, requires further investigation.

## 5 CONCLUSIONS

In summary, the present study proves a pathogenic role for mindin in renal fibrosis following renal IRI insult both in vitro and in vivo. The ability of mindin to promote renal tubulointerstitial fibrogenesis is dependent on its induction of the NF‐kB and TGF‐β/Smad pathways and the production of ECM‐related proteins. This study implies that mindin targeting may be a novel strategy for renal fibrosis treatment.

## CONFLICTS OF INTEREST

The authors declare that there is no conflict of interest regarding the publication of this paper.

## AUTHOR CONTRIBUTION

Kang Yang Wei Li, Tao Bai, Yusha Xiao and Weimin Yu participated in the design of this study. Kang Yang Wei Li and Tao Bai performed the statistical analysis. Kang Yang Yusha Xiao, Weimin Yu and Peng‐cheng Luo carried out the study and collected important background information. Kang Yang and Peng‐cheng Luo wrote the article. Pengcheng‐Luo and Fan Cheng conceived of this study and helped to draft the manuscript. All author read and approved the final manuscript.

## Supporting information

Table S1

## Data Availability

Data will be made available on request.
