# Supplementary material for: Mindin deficiency alleviates renal fibrosis through inhibiting NF‐κB and TGF‐β/Smad pathways
Source: J Cell Mol Med. 2020 Apr 6;24(10):5740–50. doi: 10.1111/jcmm.15236 (PMC7214143; doi:10.1111/jcmm.15236)
Supplement: Supplementary file 1 — Table S1 [file JCMM-24-5740-s001.doc]

Table 1. Antibody used in this study.

| Antibody | WB | IHC | IF | Company |
| --- | --- | --- | --- | --- |
| Mindin #ab171955 | 1:1000 | 1:100 | 1:50 | Abcam |
| p-p65 #3033 | 1:1000 |  | 1:50 | Cell Signaling Technology |
| p65 #ab86299 | 1:1000 | 1:100 |  | Abcam |
| p-IκBα #2859 | 1:1000 |  |  | Cell Signaling Technology |
| IκBα #4812 | 1:1000 |  |  | Cell Signaling Technology |
| Collagen I #ab138492 | 1:1000 | 1:100 |  | Abcam |
| Fibronectin #ab32419 | 1:1000 |  | 1:50 | Abcam |
| E-cadherin #3195 | 1:1000 |  |  | Cell Signaling Technology |
| p-Smad2 #18338 | 1:1000 |  |  | Cell Signaling Technology |
| Smad2 #5339 | 1:1000 |  |  | Cell Signaling Technology |
| p-Smad3 #9520 | 1:1000 |  |  | Cell Signaling Technology |
| Smad3 #9523 | 1:1000 |  |  | Cell Signaling Technology |
| Smad7 # ab216428 | 1:1000 |  |  | Abcam |
| GAPDH #ab10494 | 1:5000 |  |  | Abcam |
| Histone 3# ab176840 | 1:1000 |  |  | Abcam |
| β-actin #4970 | 1:5000 |  |  | Cell Signaling Technology |
